# Spectrum of Pathogenic Variants of the ATP7B Gene and Genotype–Phenotype Correlation in Eastern Eurasian Patient Cohorts with Wilson’s Disease

**DOI:** 10.3390/biomedicines12122833

**Published:** 2024-12-13

**Authors:** Mikhail Garbuz, Elena Ovchinnikova, Anna Ovchinnikova, Valeriya Vinokurova, Yulya Aristarkhova, Olga Kuziakova, Mariya Mashurova, Vadim Kumeiko

**Affiliations:** 1 School of Medicine and Life Sciences, Far Eastern Federal University, Vladivostok 690922, Russia; garbuzmihail.93@gmail.com (M.G.);; 2 A.V. Zhirmunsky National Scientific Center of Marine Biology Far Eastern Branch of Russian Academy of Sciences, Vladivostok 690041, Russia

**Keywords:** Wilson’s disease (WD), molecular genetic diagnostics

## Abstract

**Background/Objectives:** Wilson’s disease (WD) (OMIM 277900) or hepatolenticular degeneration is an autosomal recessive disorder caused by impaired copper excretion with subsequent accumulation in the liver, brain, and other tissues of the body. The defects in copper metabolism are based on various pathogenic variants of the ATP7B gene encoding copper-transporting P-type ATPase. The aim of this work is to search for pathogenic variants of the ATP7B gene among Eastern Eurasian patient cohorts and to pick correlations between pathogenic variants, gender, age of onset of the disease, and the course of the disease. **Methods**: The material for the study was the biomaterial of 100 people. The search for mutations was carried out by Sanger sequencing. Multiple alignment of nucleotide sequences and their analysis was performed using the MEGA-X software. To study the genotype-phenotypic correlation, an analysis of the medical records of each patient was carried out. **Results**: Most common pathogenic variant (48%) in the sample is p.His1069Gln (c.3207C>A), located in exon 14 of the ATP7B gene. Pathogenic variants of p.Glu1064Lys (c.3190G>A)—20%—and p.Met769HisfsTer26 (c.2304insC)—8%—of exons 14 and 8 were also common. For patients with pathogenic alleles p.His1069Gln (c.3207C>A) and p.Glu1064Lys (c.3190G>A), typical deviations are mental and neurological manifestations of WD. In patients with the pathogenic allele p.Met769HisfsTer26 (c.2304insC), deviations are more characteristic of the liver and a combination of various symptoms that are atypical for WD. **Conclusions**: In this study, we were able to obtain differences in symptoms in patients with different pathogenic alleles of the ATP7B gene.

## 1. Introduction

Wilson’s disease (WD) (OMIM 277900) or hepatolenticular degeneration is an autosomal recessive hereditary disorder of copper metabolism that results from various pathogenic variants in the *ATP7B* gene. The pathology was first described in 1883 by C. Westphal and A. Strumpell. The disease received its modern name—hepatolenticular degeneration—in honor of Samuel Wilson, who in 1912, having discovered degenerative changes in both the liver and the lenticular nuclei of the brain in patients with tremulous hyperkinesis, proposed calling the disease “progressive lenticular degeneration” [1].

In 1993, the *ATP7B* gene responsible for the disease was identified, localized on chromosome 13, and the mechanism for the development of the pathology was formulated. It was found that this gene encodes an enzyme, P-type ATPase, which is involved both in the excretion of excess copper from the body with bile and in the incorporation of copper into apoceruloplasmin for the synthesis of functional ceruloplasmin [2,3]. The disease manifests itself as a result of gradual accumulation of copper in the liver, brain, and tissues of other organs, leading to their damage and cell death [4]. Differences in the rate of accumulation of toxic copper in organs and individual sensitivity to their damage provide variability in the clinical manifestations of the disease. Numerous clinical observations indicated the dependence of the clinic on the age of onset, the duration of the course, and the sensitivity of tissues to copper toxicity. Forms of the disease were identified both with predominant damage to the liver, kidneys, endocrine system, and with a predominance of neurological symptoms and mental disorders [5]. Therefore, the complexity of clinical diagnostics for a long time ensured its availability only at the stage of irreversible changes in the body. Hence, WD has traditionally been considered as a rare disease, although already in 2014 P. Ferenci in his studies proved that up to 40% of children and 58% of adults already have liver cirrhosis at the time of diagnosis [6].

Patients with WD have a wide range of symptoms associated with various organs. The most common symptoms are liver dysfunction, neuropsychiatric disorders, Kaiser–Fleischer rings on the cornea, and hemolysis caused by acute liver failure.

Symptoms of WD can appear at any age [7]. An accurate diagnosis is established after a thorough assessment of the clinical picture, genetic and biochemical tests, as well as after detecting a disruption in copper metabolism. Acute liver failure often co-occurs with hemolysis [8,9]. Many biochemical tests are quite universal. For example, the ceruloplasmin level is a fairly accurate diagnostic tool for diagnosing WD [10] and can be found in the liver and neurological forms. Even in this case, one should carefully evaluate the data and adjust the results taking into account the patient’s age, habits, and lifestyle, and not exclude the possibility of similar diseases [4]. The delay in diagnosis leads to the fact that the start of therapy is years late [11,12].

WD was previously thought to be a rare disorder, but studies in several different regions have shown that the occurrence of this disease depends on the population and can occur in more than an estimated one in 35,000–45,000 people. Sequencing of the coding region of the *ATP7B* gene and adjacent splicing sites in 1000 newborns revealed a heterozygous frequency of carriers of the *ATP7B* pathogenic variants in one in 40 people, in connection with which the incidence in the UK was calculated as 1:7026 people [13]. A similar occurrence was described in France [14]. A high level of occurrence of WD is observed in regions with low population mobility and a large number of closely related populations [15]. These regions include the island of Crete, in Greece (1:15 births) [15], Kalymnos and isolated mountainous regions of Greece (1:740) [16], Romania (1:1130) [17], Croatia (1:28,000), Sardinia (1:16,700–1:34,000), Israel (1:16,000), and Costa Rica (1:19,000) [18]. Based on screening data on ceruloplasmin levels in dried blood samples from children, it was found that a high incidence of WD is observed in Japan—1:1394 [19]—and in Korea—1:3667 [20]. Due to the high incidence, as well as the potentially devastating course of the disease, it is necessary to control the prevalence of this disease in order to facilitate the diagnosis and better inform doctors, especially in regions with high incidence and low population mobility.

The incidence rate of WD within the original 1984 proposal was found in four high-quality, nationwide studies from Austria, the United States, the Czech Republic, Slovakia, Taiwan, and Hong Kong, and was estimated to range from 1:29,000 to 1:40,000 [21,22,23,24]. The lowest prevalence of WD was recorded in Sweden—1:250,000 [25].

In India, there have not been large studies examining the prevalence of WD at the national level. However, among childhood liver diseases, WD makes up 7.6–19.7%, and fifteen to twenty new cases of WD are registered annually by various neurological centers [18].

There is a large variation in the prevalence of various pathogenic variants among patients with WD depending on their geographic area and ethnic background [26]. Most reported pathogenic variants occur in single families and are rare, while some pathogenic variants are highly prevalent in certain countries and regions. For example, among the two most common pathogenic variants, p.His1069Gln (c.3207C>A) is common in Europe and North America, while p.Arg778Leu (c.2333G>T) is found in East Asia [23]. Also, common pathogenic variants characteristic of certain regions are the following: c.2007_2013del in Iceland [27], p.Met645Arg (c.1934T>G) in Spain [28], a distinct pathogenic variant of the 5′-untranslated region c.129_125del in Sardinia [29], p.Ala1135GlnfsTer13 (c.3402delC) in Southern Brazil [30], p.Cys271Ter (c.813C>A) in eastern, western, and southern India [31,32], and Gln1399Arg (c.4196A>G) in the Middle East [33]. This distribution and association with specific haplotypes is indicative of a founder effect [34].

The p.His1069Gln (c.3207C>A) pathogenic variant is the most common in Central and Eastern Europe, and therefore it has been suggested that this pathogenic variant originated in Eastern Europe [34]. The occurrence of this pathogenic variant in European countries in ascending order is observed in France (15%) [14], Turkey (17.4%) [35], continental Italy (17.5%) [29], Denmark (18%) [36], Great Britain (19%) [13], the Netherlands (33%) [37], Austria (34.1%) [9], Greece (35%) [38], Sweden (38%) [39], Romania (38.1%) [40], Hungary (42.9%) [34], former West Germany (47.9%) [41], former Yugoslavia (Slovenia, Croatia, Bosnia and Herzegovina, Serbia, Montenegro, Macedonia) (48.9%) [42], Russia (49%) [12], the Czech Republic and Slovakia (57%) [43], Bulgaria (55.8%) [44], former East Germany (63%) [41], and the highest occurrence is mentioned in Poland (72%) [45]. South of the Alps, this pathogenic variant becomes infrequent and is completely absent in Sardinia [30]. In the United States, despite ethnic diversity, the p.His1069Gln (c.3207C>A) pathogenic variant, as in European countries, was the most common (35.2%) [39]. A 2011 study in Iran also revealed that this pathogenic variant is the most common (19%) [46]. In Egypt, the p.His1069Gln (c.3207C>A) pathogenic variant ranks third in terms of occurrence (4.7%) [47].

The development of sequencing methods has led to the opportunity to study the spectrum of pathogenic variants of the *ATP7B* gene. NGS provides ~90%-accurate diagnostics. However, a delay in referring for NGS diagnostics takes up a lot of precious time. In addition, in some countries, prices for NGS diagnostics are quite high. After paying for a doctor’s appointment and other diagnostic tools, the patient’s family may not have money left for NGS. Therefore, PCR diagnostics is a fairly convenient and affordable tool for genetic screening of patients with suspected WD. In addition, it became possible to develop various alternative methods of molecular genetic diagnostics, for example, the PCR-based ARMS method [48]. The development of methods has led to a general reduction in the cost and availability of WD diagnostics for the population of any country. However, to develop a cheaper method of molecular genetic diagnostics, it is necessary to establish the spectrum of *ATP7B* gene pathogenic variants in the region being studied. Therefore, knowledge of the regional distribution of pathogenic variants is important for developing the most effective molecular screening strategies for a given region. During our study, 100 DNA samples of people with suspected WD were sequenced and, based on the data obtained, the genotype–phenotype correlation in patient cohorts with identified pathogenic variants was analyzed.

## 2. Materials and Methods

### 2.1. Materials

The materials for the study were taken from 54 patients with a previously established diagnosis of WD, 14 patients with suspected presence of this disease, and 32 first-degree relatives (a total of 100 people). The selection of patients took place in connection with the treatment of complaints of problems of a behavioral and neurological nature. The collection of blood biosamples was formed from patients of medical centers “Nevron” and “Zdorovie” after primary WD diagnosis in 2022–2024. The examination group included both patients with a preliminary diagnosis as well as long-term follow-up and therapy patients with an established diagnosis of WD.

Blood samples were obtained only from patients who signed up with informed consent. The use of human tissue materials was approved by the FEFU Ethics Committee according to Resolution №3, December 2022.

### 2.2. Methods

#### 2.2.1. Isolation of Genomic DNA

DNA extraction from whole blood samples was carried out using the ExtractDNA Blood reagent kit for isolation and purification of genomic DNA from whole blood (Evrogen, Moscow, Russia) according to the manufacturer’s protocols.

#### 2.2.2. Polymerase Chain Reaction

Before sequencing the *ATP7B* gene fragments according to Sanger, the fragments were amplified. The polymerase chain reaction (PCR) method was used to amplify certain DNA fragments. The reaction mixture for amplification of genomic DNA (20 μL) included the following: deionized water—5.5 μL; DreamTaq Green PCR Master Mix (Thermo Scientific™, Waltham, MA, USA)—10 μL; primers (10 pmol/μL)—1 μL each; DNA—2.5 μL. PCR for genomic DNA was carried out under the following conditions: preliminary denaturation at 95 °C—2 min; further for 35 cycles: 95 °C denaturation—30 s, 63 °C annealing—20 s, 72 °C synthesis—40 s; final cycle 72 °C—2 min.

The nucleotide sequences of the gene-specific primers used are shown in Table 1.

The results were visualized by 1.5% agarose gel electrophoresis with ethidium bromide (10 mg/mL, Evrogen) in TAE buffer (40 mM Tris-acetate buffer, pH 7.6, 1 mM EDTA) and photographed on the ChemiDoc MP Imaging System (BioRad, Hercules, CA, USA) in transmitted ultraviolet light. To determine the length of the fragments, a DNA marker of 1000 base pairs (bps) (Evrogen) was used.

#### 2.2.3. Purification of PCR Fragments

Purification of the PCR mixture before the BigDye reaction was carried out by precipitation with sodium acetate (C_2_H_3_O_2_Na). A total of 1.8 μL of sodium acetate was added to the PCR mixture, then 54 μL of 96% ethanol was added and left at −80 °C for 5 min. After the expiration of time, centrifugation was carried out at +4 °C at a speed of 13.4 thousand rpm for 30 min. After centrifugation, the supernatant was decanted and 100 μL of 70% ethanol was added and centrifuged again at +4 °C at a speed of 13.4 thousand rpm for 30 min. The supernatant was then poured off again and left to dry at room temperature for 15 min. At the end of the drying time, it was eluted in 20 μL of deionized water.

#### 2.2.4. Sanger DNA Sequencing

The determination of the nucleotide sequence was carried out by the classical method of direct automatic Sanger sequencing of the purified PCR product from direct primers. The reaction mixture for carrying out the BigDye reaction (10 μL) included the following: deionized water—6.25 μL; BigDye Terminator v3.1 (Thermo Scientific™)—0.5 μL; BigDye Terminator v3.1 Sequencing Buffer 5X (Thermo Scientific™)—1.75 μL; forward primer (5 pmol/μL)—0.5 μL; DNA—1 μL. The forward primers were taken from Table 1. The BigDye reaction was carried out under the following conditions: pre-denaturation at 96 °C—1 min; further for 25 cycles: 96 °C denaturation—10 s; 63 °C annealing—20 s; 60 °C synthesis—4 min.

Purification of the BigDye mixture was performed with EDTA. A total of 2.5 μL of 0.125 M EDTA (pH 8.0) and 55 μL of 96% ethanol were added to the reaction mixture, after which it was left for 20 min at −20 °C. At the end of the time, the mixture was centrifuged at a speed of 13.4 thousand rpm at +4 °C for 30 min. After centrifugation, the supernatant was decanted and 100 μL of 70% ethanol was added and centrifuged again at +4 °C at a speed of 13.4 thousand rpm for 25 min. The supernatant was then poured off again and left to dry at room temperature for 8 min. Dried samples were dissolved in 20 μL of formamide (Thermo Scientific™) and stored at −20 °C until sequencing.

The nucleotide sequence was determined by direct automatic Sanger sequencing of a purified PCR product from a forward primer. Sequencing was performed according to the manufacturer’s protocol on an ABI Prism 3100 instrument (Applied Biosystems, Waltham, MA, USA).

#### 2.2.5. DNA Sequencing Data Analysis

Analysis of the resulting nucleotide sequences was performed using the Vector NTI Advance 9.1.0 (Invitrogen, Waltham, MA, USA) and MEGA-X (MEGA Software, State College, PA, USA) programs. The search for homologous sequences for analysis was performed using the BLAST server (http://blast.ncbi.nlm.nih.gov, (accessed on 1 September 2022)). Multiple alignment of nucleotide sequences and their analysis was performed using the MEGA-X software (version 11).

#### 2.2.6. Analysis of Patient Data on Symptoms of WD

To study the genotype–phenotypic correlation, a retrospective analysis of the information contained in the patient’s case histories was carried out. Forms of manifestation of WD, the results of testing the mental and mental state of patients, MRI scans of the brain, ultrasound of the liver, and examination by an ophthalmologist using a slit lamp were taken into account. Clinical examinations were carried out at the medical centers “Nevron” and “Zdorovie”. The data obtained were analyzed using Microsoft Excel.

## 3. Results

### 3.1. Spectrum of ATP7B Pathogenic Variants

In this study, genetic material was sequenced from 68 suspected or diagnosed WD patients and 32 first-degree relatives (total of 100 people). Among the patients, six people were identified without pathogenic variants in the *ATP7B* gene, 27 patients were identified with heterozygous carriers, and 67 patients with pathogenic variants. The list and characteristics of the detected pathogenic variants of the *ATP7B* gene, as well as the number of identified alleles, are presented in Table 2.

**Figure 1:**
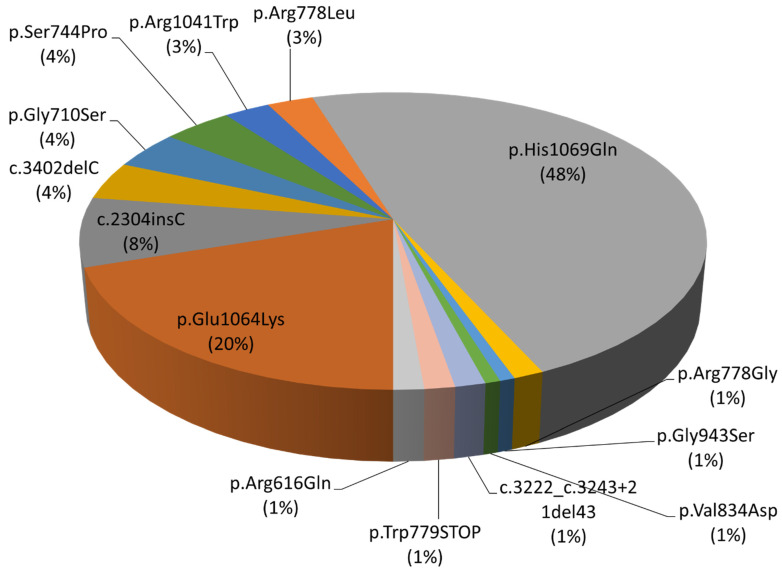
Pathogenic variant spectrum of the *ATP7B* gene.

Sanger sequencing revealed that the most common pathogenic variant (48%) in the sample is p.His1069Gln (c.3207C>A), located in exon 14 of the *ATP7B* gene. Pathogenic variants of p.Glu1064Lys (c.3190G>A)—20%—and p.Met769HisfsTer26 (c.2304insC)—8%—of exons 14 and 8, respectively, were also common (Figure 1).

The p.His1069Gln (c.3207C>A) pathogenic variant in the homozygous state was found in 28 patients (30%), while heterozygotes were detected in 16 patients (17%), including compound heterozygotes.

Among the other pathogenic variants in the homozygous state, p.Glu1064Lys (c.3190G>A) located in exon 14 was often found. In 8% of cases, single homozygous pathogenic variants p.Arg778Gly (c.2332C>G) and p.Trp779STOP (c.2336G>A) were found, located on exon 8, and c.3222_3243+21del43, found on exon 14. Also, in the study, single heterozygous pathogenic variants of p.Gly943Ser (c.2827G>A) and p.Val834Asp (c.2501T>A) located on exons 12 and 10, respectively, were encountered with a frequency of 1.4%. In addition, with a probability of 1%, one p.Arg616Gln (c.1847G>A) homozygous pathogenic variant was found in exon 5.

By their effect, the pathogenic variants were distributed as follows: SNPs leading to the replacement of an amino acid in the protein—86% (123 alleles); single deletions or insertions leading to a shift in the reading frame—12% (17 alleles); pathogenic variants that disrupt cDNA splicing—1% (two alleles); SNPs leading to a change to a stop codon—1% (two alleles).

**Figure 2:**
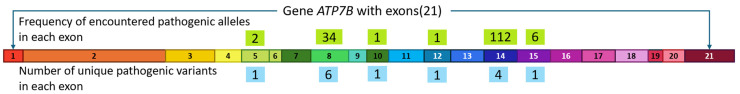
Frequency and schematic location of mutations in the *ATP7B* gene.

**Figure 3:**
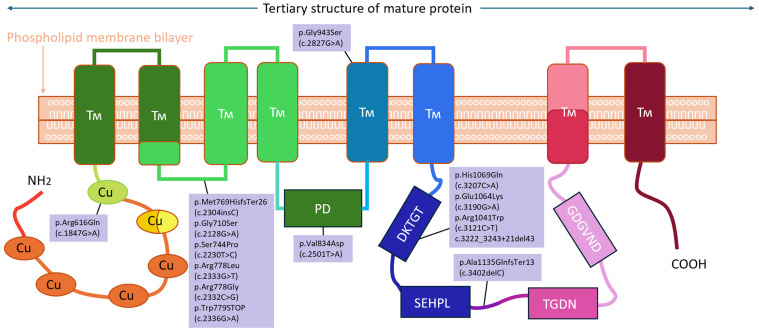
Mapping genetic variations onto the *ATP7B* protein structure.

The main variety of pathogenic variants in the *ATP7B* gene was located on exon 8 (six pathogenic variants). Exon 14 was in second place in terms of discovered unique pathogenic variants. Also, single pathogenic variants were found on exons 6, 10, 12, and 15 (Figure 2). The concentration of pathogenic variants in exons 8 and 14 and the low diversity are presumably due to the large number of relatives in this study. Exon 14 (105 alleles) ranks first in terms of the frequency of pathogenic alleles, which always contains a large number of pathogenic variants among the European population. There are eight exons (31 alleles) in the second place and 15 exons (six alleles) in the third (Figure 3).

When comparing the revealed range of pathogenic variants with pathogenic variants common in the central part of Russia, a new pathogenic allele p.Arg778Leu (c.2333G>T) was found, which is characteristic of the Asian population. The remaining pathogenic variants coincided with those described in the literature; however, the proportion of these pathogenic variants in the population differed from the data from the central part of Russia. The second most common pathogenic allele in this study was p.Glu1064Lys (c.3190G>A) with a frequency of 20%; in the literature, it is less common than 10%. In third place is the p.Met769HisfsTer26 (c.2304insC) pathogenic variant with a frequency of 7.6%; in the literature, it often ranks second with a frequency not exceeding 5%.

### 3.2. General Characteristics of Patients with WD Taken for the Study of Genotype–Phenotype Correlation

Before studying the genotype–phenotype correlation, we analyzed data regarding the gender of patients, age of onset of the disease, duration of treatment, age of onset of first symptoms, first visit to a specialist, and the region of origin of the patient. All data are presented in Table 3.

The first clinical manifestations of the disease (the debut of the disease) were observed from the age of 5 to 68 years, on average 19.6 years. However, the average age at the time of visiting a doctor is 24.5 years, which differs by 5 years from the first onset of the disease. This indicates a huge problem associated with the coverage of this disease and the consequences of late diagnosis, because after such a long period of time, patients experience irreversible physiological changes that gradually lead to the loss of a comfortable standard of living for a person and can lead to disability.

**Figure 4:**
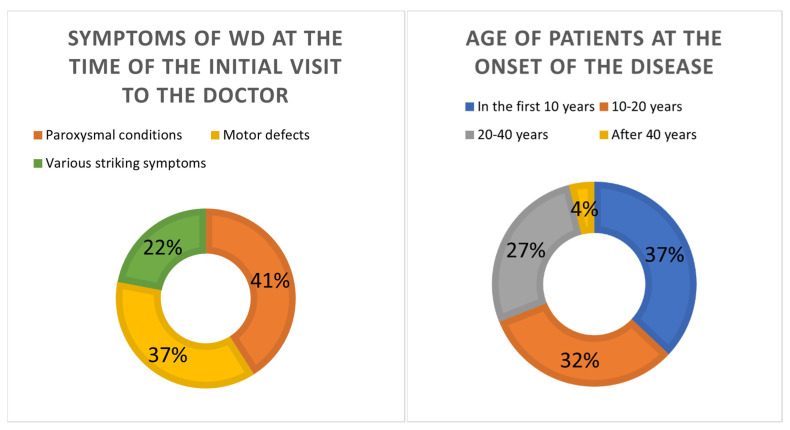
Patient cohorts according to WD manifestation.

The study found that the majority of patients with significant symptoms (69 people, 69%) fell ill during the first and second decades of life equally (37% and 32% people, respectively) and only 31 people (31%) had their first symptoms after 20 years (Figure 4). With each decade after 20 years, the number of patients seeking medical help has decreased.

As a result of the analysis of the case histories of patients, it was found that by the period of the initial visit of patients or their relatives for medical care, paroxysmal conditions predominated among neurological syndromes—41 people (41%), while the proportion of epileptic ones reached 95%. Less often, motor defects were initially manifested—37 people (37%). In addition, these occurred much less often in the early stages—22 people (22%), but the most pronounced in the clinical picture of the patients were behavioral defects, signs of mental disorders framed by somatic pathology, endocrine-hormonal disorders, skin lesions, and pathology of hematopoiesis (Figure 4).

### 3.3. Establishment of Genotype–Phenotype Correlation in Patients with WD

To simplify the comparison, patients with significant symptoms were divided into three groups: the first group included patients with the p.His1069Gln (c.3207C>A) pathogenic variant—45 people (48.9%), the second group was formed by patients with p.Glu1064Lys (c.3190G>A)—17 people (18.5%), and the third group consisted of patients with the pathogenic variant p.Met769HisfsTer26 (c.2304insC)—10 people (10.9%). The fourth group was formed from patients with rare pathogenic variants (p.Gly710Ser (c.2128G>A), p.Ser744Pro (2230T>C), p.Ala1135GlnfsTer13 (3402delC), p.Arg778Leu (2333G>T), p.Arg1041Trp (3121C>T), p.Arg616Gln (c.1847G>A), p.Arg778Gly (c.2332C>G), p.Trp779STOP (2336G>A), p.Val834Asp (2501T>A), p.Gly943Ser (2827G>A), and 3222_3243+21del43) and was taken as a group of 4–22 people (23.9%). Demographic characteristics of patients in the sample are presented in Table 4.

In the course of the analysis of examination indicators of patients, special attention was paid to the age of the patient at the stage of primary treatment for medical care, the nature of neurological manifestations at the onset of the disease, and the features of the clinical manifestations of WD at the time of diagnosis (the form of pathology, the degree of progression, and severity of the course).

Initially, the age of onset of the disease in patients was analyzed. In group 1, the maximum number of cases (40.0%) were those with the onset of the disease in the first years of life, less often (37.8%) the first symptoms occurred in the second decade (in the period from 11 to 20 years), and even more rarely, at 22.2%, they occurred in adulthood (after 20 years). The second group of patients demonstrated similar data (35%, 41%, 24%, respectively). In the patients in group 3, other ratios were observed: in the majority (50%) the disease debuted in the second decade, in the first years it occurred in 30% of cases, and in adulthood it occurred in 20% of patients in this group. The patients in group 4 did not show significant differences in the timing of the development of the first symptoms of the disease at a certain stage of life (Table 5).

As described earlier, by the time patients or their relatives first sought medical help, paroxysmal conditions (41%), motor defects (37%), behavioral defects, signs of mental disorders, and skin lesions were in evidence, with hematopoietic pathologies predominating among neurological syndromes (22%). When establishing the dependence of symptoms on pathogenic variants in patients in the first and second groups at the stage of primary treatment, motor defects predominated at 57.8% and 53%, respectively, and in the third and fourth groups these symptoms took second place with a frequency of 20% and 32%. In the third and fourth groups, the first place was occupied by paroxysmal disorders with a frequency of 50% and 59% respectively, and in the first and second groups, these symptoms occurred only in 35.5% and 41%. Mixed symptoms were not widespread in any of the groups but were much more common in group 3 (30%) than in the others (6.7% in group 1, 6% in group 2, 9% in group 4).

Representatives of each group showed changes on MRI of the brain. As the pathology spread to the structures of the midbrain, the phenomenon of the so-called “giant panda face” was formed. In representatives of group 1, changes were recorded in more than half of the cases (68.8%). In group 2, the incidence was slightly lower and changes on MRI were recorded in 58.8%. Changes on MRI in the third and fourth groups were significantly lower—30% and 32%.

Deterioration of the liver is common in patients with WD, and patients underwent liver ultrasound to look for abnormalities. The liver pathology detected by ultrasound was represented by a slight increase in size and unpronounced structural changes. Most often, liver defects were observed in the third and fourth groups—60% and 55%, less often in the first group—46.7%, and much less often in the second group—41%.

**Figure 5:**
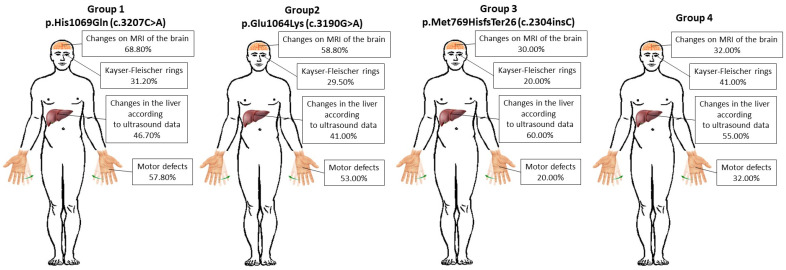
Schematic representation of the frequency of occurrence of symptoms in each group.

WD is also characterized by copper deposits along the periphery of the cornea, called Kayser–Fleischer rings. This sign and its elements were most common in the fourth group—41%, less common in the first and second groups—31.2% and 29.5%, and least common in the third group—20% (Figure 5).

To detect severe impairment of mental, intellectual, and cognitive functions among patients with WD, tests, examinations by doctors, observations over a long period of time, as well as a survey of the next of kin about the daily behavior and life of patients, were carried out. The greatest deviations were observed in the first and second groups—26.6% and 23.8%, the fourth group took second place—14%, and the smallest number of such patients was in the third group—2% (Table 5).

Summarizing the results of the study of genotype–phenotypic correlation, it should be noted that for patients in the first and second groups (p.His1069Gln (c.3207C>A) and p.Glu1064Lys (c.3190G>A)), mental and neurological manifestations of WD are common deviations; in patients in the third group (p.Met769HisfsTer26 (c.2304insC)) deviations are more typical for the liver and a combination of various symptoms atypical for WD.

The first and second groups are characterized by hyperkinesis, brain changes, the presence of Kayser–Fleischer rings, and severe impairments of mental, intellectual, and cognitive functions; also, in patients with these pathogenic variants, the disease debuted in the first 20 years of life, then the detection rate decreased. In general, the neurological and psychiatric symptoms of WD have low mortality, but patients with these forms of manifestations are more likely to experience irreversible consequences of the disease. It is also more difficult for such patients to lead a normal life and interact with society.

The third group of patients is characterized by symptoms such as paroxysmal conditions, liver changes on ultrasound, and other symptoms uncharacteristic of WD. In patients with these pathogenic variants, the onset of the disease is often delayed by up to 10–20 years.

The fourth group was characterized by paroxysmal conditions, changes in liver on ultrasound, and Kayser–Fleischer rings.

## 4. Discussion

The study of Wilson’s disease spans many areas of biology and medicine. However, the role of copper in the cell and the body, the ways of removing copper from the body, and the causes of WD are still not fully known. Also, in recent years, many researchers from different parts of the world have reported that this disease is much more common than is mentioned in the literature (1:30,000 people) [13,14,49]. For example, in recent studies of the occurrence of WD in western Russia, the estimated frequency was 1:10,000 (confidence interval from 1:12,500 to 1:8333), and heterozygous carriers were estimated as 1:50 (confidence interval from 1:63 to 1:42) [12]. Such a huge difference in numbers indicates a flaw in the established diagnostic system for this disease, since even characteristic biochemical markers can give dubious results that delay diagnosis, and this is also affected by doctors’ poor awareness about this disease. A delay in making a diagnosis can lead to irreversible changes in the human body, early disability, loss of ability to work and dropping out of society, as well as death. However, timely treatment can almost completely relieve the patient of WD symptoms [4,5]. To summarize the above, it is worth emphasizing the need to develop a method for molecular genetic diagnosis of this disease that is simple and accessible to the general public.

WD is caused by defects in the *ATP7B* gene. To date, more than 800 variations of pathogenic variants have been found that lead to various deviations in the maturation and functioning of the protein [11]. A defective protein causes disruption of many cellular processes: the cell cycle, redox processes, the exchange of other metals (iron, zinc, magnesium, etc.), epigenetic processes, and much more. Symptoms and age of onset can also be influenced by external factors (environment, diet, alcohol consumption, etc.) and the personal characteristics of the patient’s body (epigenetics, other congenital diseases); however, the range of changes in the body with WD depends on the degree of protein defects; that is, it depends on pathogenic variants in the *ATP7B* gene [50,51]. Thus, by collecting data on the genotype–phenotype correlation of WD in the future, it will be possible to make a forecast regarding the degree of manifestation and the nature of symptoms. Russian scientists are searching for the most common pathogenic variants of the *ATP7B* gene using methods based on PCR or NGS sequencing of the entire gene. The list of pathogenic variants identified by PCR-based methods is constantly growing. For example, about 15 years ago, a search was carried out for only two common pathogenic variants in the *ATP7B* gene; after 2 years, their number increased to eight. At the moment, diagnostics for 12 pathogenic variants have been developed at the Medical Genetic Research Center. Also, molecular genetic analysis of the *ATP7B* gene is carried out in laboratories in the cities of St. Petersburg, Omsk, and Novosibirsk [12,52]. However, NGS sequencing of the entire gene or part of it is an expensive diagnostic method, which is often inaccessible to the average person. However, genetic diagnosis before the appearance of irreversible changes, or as part of family screening, is necessary for a wide mass of people, and therefore should be cheap and accessible. The best option in this case is to develop methods based on PCR with a wide range of detectable pathogenic variants and easy to use.

The study material included 68 patients with suspected WD or a previously established diagnosis and 32 first-degree relatives (100 people in total). Patients for genetic testing were residents of the Russian Far East, regardless of gender, age, and nationality. The examination group included both patients with a preliminary diagnosis and patients with an established diagnosis of WD who were observed and receiving therapy for a long time.

In the study group, there was no significant dependence of WD on gender. The bulk of the patients were descendants of immigrants from regions bordering Eastern Europe, Ukraine, Belarus, and Poland—65%. In second place were the descendants of immigrants from the central part of Russia and Siberia—29%.

Sanger sequencing revealed that, with a frequency of 48%, the most common pathogenic variant in the Russian Far East is p.His1069Gln (c.3207C>A), located in exon 14 of the *ATP7B* gene, which is generally consistent with data from European and domestic studies [12,34]. In second place in occurrence with a frequency of 20% is the p.Glu1064Lys (c.3190G>A) pathogenic variant, also located in exon 14. According to European and most domestic data, this pathogenic variant is not included in the list of frequently occurring ones [12,34]; however, there are domestic data refuting this for the European part of Russia [12]. The third place in occurrence with a frequency of 8% was occupied by the p.Met769HisfsTer26 (c.2304insC) pathogenic variant, located on exon 8. This pathogenic variant is often found not only in Europe and the European part of Russia, but also in some Asian countries [30,35]. The most common pathogenic variants occurred in exons 8 and 14—94%. This fact is presumably due to the large number of relatives in this study.

When examining the patients’ relatives, it was possible to detect 22 heterozygous carriers, one compound heterozygous form, and six homozygous carriers. The study also analyzed material from 16 patients with suspected WD. A feature of these patients was the difficulty of diagnosis due to atypical and rare symptoms for WD, as well as biochemical parameters that were within normal limits. Among these patients, nine homozygotes were identified, including those with rare pathogenic variants, four heterozygotes, and no pathogenic variants were found in three patients. Subsequently, symptomatic patients were prescribed copper-eluting therapy depending on the severity of the disease. This helped relieve some symptoms and improve the condition of patients. Patients who have not yet developed WD were prescribed recommendations from doctors and periodic examination by a specialist. The fact that when examining relatives, a large number of patients suffering from copper metabolism disorders with different variants of pathogenic forms, as well as diagnostic problems based only on phenotypic manifestations and biochemical data, speaks of the importance of accessible preventive molecular genetic diagnostics.

This study identified six patients without pathogenic variants in the *ATP7B* gene but with severe symptoms of WD. This discrepancy has already been described in the literature; in approximately 10% of patients with pronounced symptoms of WD, pathogenic variants are not detected. Previously, this was associated with extended intragenic deletions, insertions, or duplications that are not recognized by sequencing [53]. In recent years, researchers have often suggested that the course of the disease and symptoms can also be influenced by pathogenic variants in introns, which affect the process of protein maturation, leading to its defectiveness; research on this topic is currently underway [54].

Subsequently, to simplify comparison, patients with pathogenic variants were divided into four groups: group 1 included patients with the p.His1069Gln (c.3207C>A) pathogenic variant—45 people (48.9%), group 2 included patients with p.Glu1064Lys (c.3190G>A) pathogenic variants—17 people (18.5%), and group 3 consisted of patients with the p.Met769HisfsTer26 (c.2304insC) pathogenic variant—10 people (10%). The last group was taken as a fourth group and included patients with rare pathogenic variants. It included 22 people (23.9%) with the following pathogenic variants: p.Gly710Ser (c.2128G>A), p.Ser744Pro (c.2230T>C), p.Ala1135GlnfsTer13 (c.3402delC), p.Arg778Leu (c.2333G>T), p.Arg1041Trp (c.3121C>T), p.Arg616Gln (c.1847G>A), p.Arg778Gly (c.2332C>G), p.Trp779STOP (c.2336G>A), p.Val834Asp (c.2501T>A), p.Gly943Ser (c.2827G>A), and c.3222_3243+21del43.

As a result of studying medical histories, symptoms, analyses, and tests, it was found that patients with the p.His1069Gln (c.3207C>A) pathogenic variant are more likely to have motor defects, brain changes, the presence of Kayser–Fleischer rings, and severe impairment of mental, intellectual, and cognitive functions. Also, in patients with this pathogenic variant, the disease debuted more often before the age of 10, and subsequently the detection rate decreased. Patients with the p.Glu1064Lys (c.3190G>A) pathogenic variant are also characterized by motor defects, brain changes, the presence of Kayser–Fleischer rings, and severe impairment of mental, intellectual, and cognitive functions, but the onset of the disease more often occurred in the second decade of life. Patients with the p.Met769HisfsTer26 (c.2304insC) pathogenic variant were more likely to show hyperkinesis and liver changes on ultrasound. The onset of the disease was often delayed until 10–20 years. The fourth group was characterized by paroxysmal conditions, changes on liver ultrasound, and Kayser–Fleischer rings.

The most frequent pathogenic allele in the European population, p.His1069Gln (c.3207C>A), was also the most frequent in our cohort [55]. Mild disease course, neurological symptoms, and longer survival have been described for this variant [55,56,57,58]. In our study, carriers of this variant also predominantly had neurological and psychiatric abnormalities, as well as MRI changes. Nonsense truncations of the protein, frame shifts, or splice site variants have a significant functional and structural effect on the *ATP7B* protein and can be associated with more severe disease (early onset, low ceruloplasmin levels, high liver copper content). In contrast, missense variants are associated with late onset, less severe disease, and more neurological manifestations [6,45,59,60]. However, contrary to this, the third most frequent pathogenic allele was frame shift p.Met769HisfsTer26 (c.2304insC). Other studies of pathogenic variants from exon 8 have demonstrated a later onset of WD [61]. In our cohort, this is also typical for p.Met769HisfsTer26 (c.2304insC).

## 5. Conclusions

Establishing a genotype–phenotype correlation of WD is a complex task since the full list of factors influencing the course and onset of the disease has not yet been fully identified. In addition, such studies often examine biochemical parameters of patients or take individual parameters that can be combined into one group. In our study, we attempted to generalize the symptoms and combine them into groups to simplify the analysis. As a result, we were able to obtain a difference in symptoms in patients with different pathogenic alleles of the *ATP7B* gene despite a relatively small sample. Further studies will be aimed at expanding the sample and optimizing the comparison of symptoms.

## Figures and Tables

**Table 1:** Primers for exon amplification of the *ATP7B* gene.

Exon	Forward Primer	Reverse Primer
2A	CATTGTTTTCCATTTTCTCAGTG	CCGATGGCACATATTTCACAG
2B	CTTGCCAGTCATGTGTGAAGT	CAGAAGATAAAGGTCTCTTTGG
2C	GATATTGAGCGGTTACAAAGCA	ACTCCCTTCGGCTCCATC
2D	GACCCTTCTTGTACCAGCC	CTCACCTATACCACCATCC
3	GACAATGAACCCTCACCAAG	GAATACGAGGTCTATACGCAG
4	CCACCCAGAGTGTTACAGC	CTAATCACAAAGATGGATGTGTC
5	CTGCCTGTTACCTAGACTC	CATCTTTCTCTTACCCATTCAC
6	CCCACAAAGTCTACTGAGG	GCTAATCCAGGAGGAAGGC
7	CTTAAACTGTGTCCTCAGAAG	GGAAGGGAGAGGTCTGC
8	GCCCATCACAGAGGAAGAA	GAGATTTGTTTACTGAAGGAGC
9	GGTGGATAGCAAGTAACGC	GAGAGTGGTGATCTTACTGTG
10 + 11	GCAAATACAGTGTAACTATTGTAAC	CCCAGAACTCTTCACATAATTTC
12	CCAATCTTTATCCATGCTTGTG	GTGACTGTTTATCCTACTCTG
13	CCTTATTGAACTCTCAACCTG	CTCTGTTGCTACTGTTGTTATTC
14	CTGAGATTGAACGACAGAGG	GTGAGGAATAAAAGAGCATTGG
15	CGCTTTCCGCTGCTCTC	CAGAGGCAATCACTGCTGG
16	CACAAGAGGTGCTTACAAGG	CTGAAATTAAGAGAGGAAGGC
17	CACTCGTAATCCTATTCCTTG	GGAGTACAGCTCAGTGCTG
18 + 19	GCTGCTATCTGATACCTTTTG	CTGGGAGACAGAAGCCTTT
20	GAACATCAGGGCGAGTGG	GAATGGGAAATGAGAGGCAAG
21A	GAATGGCTCAGATGCTGTTG	GCAGGATGACTGGACATATC
21B	CAGGGATGAGGAGCAGTAC	GCAGGGTTGCGAGGGG
21C	GTACATAGTCTGTTCCTTTTCTC	CACGAGGTGACAGTCAGAA
21D	CATGGGAAACGTATGTGTGC	GGTTGGTTGGGAGGATTAGA
21E	GGATTAAATTCTGGGTGAAACC	GGCTGAAAACAAGGAAAACACA
21F	CCTTCACCTTGGATGGTTAG	CTGACCACGCATCTCACTC

**Table 2:** List and allelic frequencies of *ATP7B* gene pathogenic variants identified in patients with suspected or diagnosed WD.

Exon	Protein Domain	Position in cDNA	Position in Protein	Pathogenic Variant Effect	Number of Alleles
5	Transmembrane	c.1847G>A	p.Arg616Gln	Loss of function	2 (1%)
8	Transmembrane	c.2304insC	p.Met769HisfsTer26	Frame shift	11 (8%)
		c.2128G>A	p.Gly710Ser	Loss of function	6 (4%)
		c.2230T>C	p.Ser744Pro	Loss of function	6 (4%)
		c.2333G>T	p.Arg778Leu	Loss of function	4 (3%)
		c.2332C>G	p.Arg778Gly	Loss of function	2 (1%)
		c.2336G>A	p.Trp779STOP	Stop codon formation	2 (1%)
10	Transmembrane	c.2501T>A	p.Val834Asp	Loss of function	1 (1%)
12	Transmembrane	c.2827G>A	p.Gly943Ser	Loss of function	1 (1%)
14	ATP-binding	c.3207C>A	p.His1069Gln	Loss of function	70 (48%)
		c.3190G>A	p.Glu1064Lys	Loss of function	29 (20%)
		c.3121C>T	p.Arg1041Trp	Loss of function	4 (3%)
		c.3222_3243+21del43	-	Protein maturation disorder	2 (1%)
15	ATP-binding	c.3402delC	p.Ala1135GlnfsTer13	Frame shift	6 (4%)

**Table 3:** Demographic characteristics of patients with WD.

Total number of patients analyzed	100	
Patient gender	Male	52 (52%)
	Female	48 (48%)
Average age of patients at the time of visiting a doctor	24.5 years	
Average age of onset of WD	19.6 years	
Average length of observation with a doctor	8.9 years	
Patient’s region of origin	Descendants of immigrants from regions bordering Eastern Europe, Ukraine, Belarus, Poland—65% Descendants of immigrants from central Russia and Siberia—29% Asian patients—4% Immigrants from Azerbaijan—2%	

**Table 4:** Demographic indicators of patients with significant symptoms divided into different groups.

Indicators		p.His1069Gln (c.3207C>A)	p.Glu1064Lys (c.3190G>A)	p.Met769HisfsTer26 (c.2304insC)	4th Group
Patient gender	Male	48.89%	52.94%	30%	68.18%
	Female	51.11%	47.06%	70%	31.82%
Average age of patients at the time of the study		17.6	19.3	26.2	34.7
Region of residence		Primorsky Territory—62.2%; Khabarovsk Territory—22.2%; Kamchatka—8.9%; Sakhalin—6.7%.	Primorsky Territory—52.9%; Khabarovsk Territory—29.4%; Kamchatka—5.9%; Sakhalin—11.8%.	Primorsky Territory—20%; Khabarovsk Territory—0%; Kamchatka—50%; Sakhalin—30%.	Primorsky Territory—36.4%; Khabarovsk Territory—31.8%; Kamchatka—13.6%; Sakhalin—18.2%.

**Table 5:** Distribution into groups of patients with WD selected to establish the genotype–phenotype correlation. Common symptoms are marked in green, and rare symptoms are shown in red. SD—standard deviation. The *p*-values were adjusted with the Benjamini–Hochberg method.

Symptoms		p.His1069Gln (c.3207C>A)	p.Glu1064Lys (c.3190G>A)	p.Met769HisfsTer26 (c.2304insC)	4th Group
Age of disease onset ± SD (*p* = 0.86)	First 10 years	40.00 ± 4.36%	35.00 ± 1.53%	30.00 ± 1.53%	32.00 ± 0.58%
	10–20 years	37.80 ± 4.36%	41.00 ± 1.53%	50.00 ± 1.53%	32.00% ± 0.58%
	After 20 years	22.20 ± 4.36%	24.00 ± 1.53%	20.00 ± 1.53%	36.00% ± 0.58%
Primary symptoms ± SD (*p* = 0.09)	Paroxysmal conditions	35.50 ± 1.53%	41.00 ± 4.16%	50.00 ± 1.53%	59.00 ± 5.51%
	Motor defects	57.80 ± 1.53%	53.00 ± 4.16%	20.00 ± 1.53%	32.00 ± 5.51%
	Other symptoms	6.70 ± 1.53%	6.00 ± 4.16%	30.00 ± 1.53%	9.00 ± 5.51%
Changes on MRI of the brain ± SD (*p* = 0.59)		68.80 ± 12.23%	58.80 ± 12.23%	30.00 ± 12.23%	32.00 ± 12.23%
Changes in the liver according to ultrasound data ± SD (*p* = 0.59)		46.70 ± 7.09%	41.00 ± 7.09%	60.00 ± 7.09%	55.00 ± 7.09%
Kayser–Fleischer rings ± SD (*p* = 0.59)		31.20 ± 5.48%	29.50 ± 5.48%	20.00 ± 5.48%	41.00 ± 5.48%
Severe impairment of mental, intellectual, and cognitive functions ± SD (*p* = 0.59)		26.60 ± 4.72%	23.80 ± 4.72%	2.00 ± 4.72%	14.00 ± 4.72%

## Data Availability

Data are contained within the article. Detailed data are available upon request from the first author.
